# *Mycobacterium avium* subsp. *paratuberculosis* in an Italian Cohort of Type 1 Diabetes Pediatric Patients

**DOI:** 10.1155/2012/785262

**Published:** 2012-07-11

**Authors:** Maria Luisa Manca Bitti, Speranza Masala, Francesca Capasso, Novella Rapini, Simona Piccinini, Federica Angelini, Andrea Pierantozzi, Roberta Lidano, Silvia Pietrosanti, Daniela Paccagnini, Leonardo A. Sechi

**Affiliations:** ^1^Pediatric Diabetology Unit, Policlinico di Tor Vergata, University of Rome Tor Vergata, 00133 Rome, Italy; ^2^Dipartimento di Scienze Biomediche, Università di Sassari, 07100 Sassari, Italy; ^3^Department of Pediatrics, University of Rome Tor Vergata, Children's Hospital Bambino Gesù, Piazza Sant'Onofrio 4, Rome, Italy; ^4^Division of Pediatrics, Department of Public Health and Cell Biology, University of Rome Tor Vergata, 00165 Rome, Italy; ^5^Unité d'Immuno-Allergologie et Rhumatologie, Département Médico-Chirurgical de Pédiatrie, Centre Hospitalier Universitaire Vaudois (CHUV), Rue du Bugnon 21, 1011 Lausanne, Switzerland; ^6^Department of Internal Medicine, University of Rome Tor Vergata, 00133 Rome, Italy

## Abstract

*Mycobacterium avium subsp. paratuberculosis* (MAP) is the etiological agent of Johne's disease in ruminants. Recent studies have linked MAP to type 1 diabetes (T1D) in the Sardinian population. The aim of this study was to investigate the prevalence of MAP infection in a T1D cohort from continental Italy compared with healthy control subjects. 247 T1D subjects and 110 healthy controls were tested for the presence of MAP. MAP DNA was detected using IS900-specific polymerase chain reaction (PCR). The presence of antibodies towards a MAP antigen, heparin binding hemoagglutinin (HBHA), was detected by ELISA. We demonstrated a higher MAP DNA prevalence in plasma samples from T1D patients and a stronger immune response towards MAP HBHA, compared with healthy control subjects. Moreover, in the recent onset patients, we observed an association between anti-MAP antibodies and HLA DQ2 (DQA1 0201/DQB1 0202). These findings taken together support the hypothesis of MAP as an environmental risk factor for the development of T1D in genetically predisposed subjects, probably involving a mechanism of molecular mimicry between MAP antigens and pancreatic islet **β**-cells.

## 1. Introduction

Type 1 diabetes (T1D) is an autoimmune endocrine disorder characterized by T cell infiltration of the pancreas and production of autoantibodies [1]. Several loci, primarily HLA, and environmental factors might be responsible for the disease manifestation [2, 3]. Indeed, the clinical onset of T1D is described as a sequence of multiple environmental insults in genetically susceptible subjects [3].

It is common knowledge that epitope mimicry is the mechanism by which an infectious agent exposing epitopes immunologically very similar to the antigenic determinants of the host induces a loss of tolerance to self antigens, triggering an autoimmune response [4, 5]. The epidemiological data suggest that some viruses such as enteroviruses, and more specifically coxackieviruses, but also rotaviruses, cytomegalovirus, parvovirus, and encephalomyocarditis virus might have a role in the T1D pathogenesis [3]. To a lesser extent, also bacteria have been indicated as environmental trigger of T1D onset. Direct evidence exists in rodents, that indeed diabetes is aggravated under specific pathogen-free conditions or upon administration of antibiotics [6]. In other studies, however, administration of antibiotics prevented diabetes [7, 8].

Recently, *Mycobacterium avium* subsp. *paratuberculosis* (MAP) has been proposed as a new environmental trigger that might contribute to T1D pathogenesis [9, 10]. MAP causes a chronic granulomatosis enteritis, known as Johne's disease, in ruminants [11]. This pathogen is characterized by the ability to survive pasteurization and chlorination [12], so that it can be detected in milk and dairy products obtained from infected ruminants, that are asymptomatic reservoir [11, 12]. It is well known that in Sardinia MAP infection is endemic in sheep husbandry and that this pathogen is associated with Crohn's disease [13–15], suggesting that MAP could be an environmental factor [16, 17]. MAP infection is highly prevalent in T1D patients in Sardinia, one of the regions with the highest T1D incidence worldwide. Indeed, MAP DNA was isolated from blood in 63% of Sardinian T1D patients, but only in 16% of healthy controls [9]; the MAP envelope protein MptD can be detected in the blood of 47% Sardinian T1D patients, but in a smaller proportion of type 2 diabetes (T2D) patients (8%) and healthy controls (13%) [16, 18]; and MAP bacilli can be cultured from blood [16].

In addition, recent studies on Sardinian population have demonstrated an association between MAP and multiple sclerosis [19, 20], extending its role as environmental trigger of different autoimmune diseases. 

We could confirm the association between MAP and T1D in a cohort of children from continental Italy, evaluating the presence of MAP DNA and of anti-MAP antibodies in the sera of patients and healthy subjects.

## 2. Materials and Methods

### 2.1. Patient and Control Sera Samples

A total of 357 participants comprising of 247 with T1D and 110 healthy controls, attending the Pediatric Diabetes Unit of Tor Vergata University Hospital of Rome, were tested for the presence of MAP. Blood from patients was centrifuged, and serum supernatants were used for enzyme-linked immunosorbent assay (ELISA); the remaining sera were aliquoted and stored frozen at −20°C for short-term storage (<6 months) and −80°C for long-term storage (>6 months). A second blood sample was used to collect PBMCs for DNA extraction. Written informed consent to participate to the study was obtained from all subjects or from their parents, according to the Institutional Ethical Committee.

### 2.2. Protein Expression and Purification

MAP heparin binding haemagglutinin was purified as described earlier [21] The HBHA was subcloned in pET15 (Novagen Inc., Madison, WI), and the recombinant histidine-tagged protein was purified by nickel chromatography according to the standard protocols [21].

### 2.3. MAP IS900 Amplification

 The presence of MAP-specific IS900 signature using total DNA extracted from PBMCs was performed as previously published [9, 14]. Different amplicons obtained by the second-round nested PCR were sequenced to confirm IS900 identity.

### 2.4. ELISA

An indirect ELISA to detect antibodies anti-MAP HBHA was performed as described previously [21]. ELISA was performed in 96-well microplates (Nunc-Immuno plate). Purified HBHA protein was diluted in carbonate bicarbonate buffer (Sigma-Aldrich) at a final concentration of 5 *μ*g/mL and used as an antigen. Each well was coated with 50 *μ*L of diluted antigen overnight at 4°C. The next day, the unabsorbed antigen was discarded, and wells were blocked with 5% nonfat-dried milk (Sigma-Aldrich) at 37°C for 1 h. Plates were washed three times with 200 *μ*L phosphate-buffered saline-Tween (PBS-T) (PBS-0.05% Tween 20) before 100 *μ*L of diluted serum (1 : 100 in PBS-T) was added to each well. After 2 h, plates were washed five times with PBS-T and incubated for 1 h with 100 *μ*L of anti-human immunoglobulin G alkaline phosphatase antibody (Sigma-Aldrich) diluted 1 : 1,000 in PBS-T. Five rounds of washing were performed, and 200 *μ*L of Sigma Fast p-nitrophenyl phosphatase substrate was added to each well. As the color developed, plates were read at 405 nm on a VERSA Tunable Max microplate reader (Molecular Devices). Cut-off was set at 0.67 and calculated as described previously [15, 18, 21].

### 2.5. Statistical Analysis

The associations of the frequencies were evaluated by Fisher's exact (2 × 2 tables) and chi-square tests. A *P*-value ≤ 0.05 was considered statistically significant. Analyses were performed using SPSS 13.0 (SPSS, Inc., Chicago, IL).

## 3. Results 

We evaluated the presence of MAP DNA in the PBMCs of 247 T1D patients and 110 healthy subjects. We found that MAP DNA (IS900 PCR) was detected in the sera of 30 T1D patients (12.1%) and 5 healthy controls (4.5%). Our results (Table 1) indicated that the presence of MAP DNA is significantly associated with T1D (*P* = 0,033). Anti-HBHA antibodies (HBHA is a membrane MAP antigen involved in virulence) were also searched by ELISA. We tested the sera of 247 T1D patients and 110 healthy controls and the results, expressed as optical density (OD), are reported in Table 2. The HBHA antigen gave strong ELISA values (cut-off titer value of 0.67) in 76 patients (30.8%) but only in 5 healthy subjects (4.5%). These findings confirm the strong association between the presence of anti-MAP antibodies and T1D (*P* < 0.0000). Interestingly, only in T1D patients sera, a positivity of both MAP DNA and antibodies anti-MAP was observed (*P* = 0,0000, Table 3). Considering the high frequencies of MAP antibodies positive subjects, we analyzed by chi-square test the association between these values and the different parameters that characterized our cohort and we did not find significant association with any of the variables investigated (data not shown). When we stratified our cohort in 40 patients with newly diagnosed T1D (within six months after the onset) and in 182 patients 6 months after the onset, we did not observe any difference in MAP DNA and antibodies anti-MAP positivity between the two groups (data not shown). Interestingly, in the newly diagnosed T1D patients group, we found a significant correlation between the antibodies anti-MAP positivity and the presence of HLA DQA1∗0201/DQB1∗0202 (Table 4). 

## 4. Discussion

MAP is the etiological agent of paratuberculosis, a chronic granulomatosis enteritis in ruminants [11]. The bacteria is widely disseminated and it can be detected in milk and dairy products [12].

Originally, MAP has been associated with immune related disorders, such as Crohn's disease [13, 14, 17, 22]. Recently, different works suggested that MAP may be considered a bacterial risk factor involved in the development of T1D, but this relationship was demonstrated only within the Sardinian population [9, 10, 18, 21, 23, 24]. 

Here, we showed that an association between MAP and T1D was observed also in a cohort of patients from continental Italy, with a genetic background different from Sardinians. In detail, we showed a statistical significant association between the presence of MAP DNA and T1D and also a strong association with the presence of anti-MAP antibodies. These findings support the hypothesis that MAP might have a potential role as environmental trigger of T1D. To further test this view, we compared the prevalence of the positivity for MAP DNA and anti-MAP antibody in patients at onset and with a long-term disease, and we did not find significant differences. However, due to the numerical imbalance between the two groups, we believe that this data does not reflect the real distribution of MAP frequencies in the whole patient population. It would be interesting to perform a targeted study that might specifically address the question whether MAP infection is temporally related to the onset of the disease. The fact that only in T1D patients sera a positivity of both MAP DNA and antibodies anti-MAP was observed could link even more T1D and MAP since HBHA homologue are present also in other mycobacteria [21] and ELISA positivity (but negative PCR) in healthy control could reflect a cross-reaction with environmental mycobacteria. It is also true that PCR and ELISA have different sensitivity and search for different things: IS900 MAP-specific PCR positivity reflects the presence of MAP specific DNA, ELISA positivity reflects a humoral response toward MAP but the host could be actually MAP free. 

Interestingly, we showed an association between anti-MAP antibodies and HLA DQ2 (0201/0202) in patients at onset. We might speculate that this HLA could be involved in the MAP recognition, favouring its role as an environmental trigger. Indeed, it is known that some HLA typing may promote molecular mimicry between microbial proteins and islet autoantigens [2]. In line with this hypothesis, another MAP protein, namely, MAP3865c, displays a sequence homology with the *β*-cell antigen zinc transporter 8 (ZnT8) [25], which is targeted by aAbs in T1D patients [26].

In conclusion, we report for the first time the association between T1D and MAP in a population outside Sardinia, suggesting that there is sufficient indirect evidence to warrant a focus on MAP as potential trigger in T1D. 

## Figures and Tables

**Table 1:** Distribution of IS900 PCR (MAP genome) detection in a cohort of Type 1 Diabetes (T1D) patients compared to healthy controls (CTR). Percentage and absolute values.

	PCR IS900		Total	*P* value
	Negative	Positive		
T1D	87,9% (*n* = 217)	12,1% (*n* = 30)	100% (*n* = 247)	*P* < 0,033
CTR	95,5% (*n* = 105)	4,5% (*n* = 5)	100% (*n* = 110)	

**Table 2:** HBHA antigen (expressed as OD values) positivity in a cohort of Type 1 Diabetes patients (T1D) compared to healthy controls (CTR). Percentage and absolute values.

	OD		Total	*P* value
	<0,67	≥0,67		
T1D	69,2% (*n* = 171)	30,8% (*n* = 76)	100% (*n* = 247)	*P* < 0,0000
CTR	95,5% (*n* = 105)	4,5% (*n* = 5)	100% (*n* = 110)	

**Table 3:** Combination of distribution (by chi-square test) of IS900 PCR (MAP genome) and HBHA MAP antigen in a cohort of Type 1 diabetes patients (T1D) compared to healthy controls (CTR). Percentage and absolute values.

	Combination (PCR versus HBHA antigen)				Total	*P* value
	0-0	1-0	0-1	1-1		
T1D	60,7% (*n* = 150)	27,1% (*n* = 67)	8,5% (*n* = 21)	3,6% (*n* = 9)	100% (*n* = 247)	*P* < 0,0000
CTR	90,9 (*n* = 100)	4,5% (*n* = 5)	4,5% (*n* = 5)	0% (*n* = 0)	100% (*n* = 110)	

0: negative, 1: positive.

**Table 4:** Association between the positivity of anti-MAP antibodies and the presence of HLA DQA1∗0201/DQB1∗0202 in a group of patients with newly diagnosed T1D (onset) compared to a group of patients 6 months after the onset (not onset).

	HBHA (OD)	DQ2 (0201/0202)		Total	*P* value
		Negative	Positive		
Onset	0	92,6% (*n* = 25)	7,4% (*n* = 2)	100% (*n* = 27)	*P* < 0,0000
	1	61,5% (*n* = 8)	38,5% (*n* = 5)	100% (*n* = 13)	

Not onset	0	84,7% (*n* = 105)	15,3% (*n* = 19)	100% (*n* = 124)	*P* = ns
	1	77,6% (*n* = 45)	22,4% (*n* = 13)	100% (*n* = 58)	

HBHA OD: 0 < 0,67, 1 ≥ 0,67.

## References

[1] Taplin C, Barker J (2008). Autoantibodies in type 1 diabetes. *Autoimmunity*.

[2] Concannon P, Rich SS, Nepom GT (2009). Genetics of type 1A diabetes. *New England Journal of Medicine*.

[3] Van Belle TL, Coppieters KT, Von Herrath MG (2011). Type 1 diabetes: etiology, immunology, and therapeutic strategies. *Physiological Reviews*.

[4] Davies JM (1997). Molecular mimicry: can epitope mimicry induce autoimmune disease?. *Immunology and Cell Biology*.

[5] Sfriso P, Ghirardello A, Botsios C (2010). Infections and autoimmunity: the multifaceted relationship. *Journal of Leukocyte Biology*.

[6] Bach JF (2002). The effect of infections on susceptibility to autoimmune and allergic diseases. *New England Journal of Medicine*.

[7] Brugman S, Klatter FA, Visser JTJ (2006). Antibiotic treatment partially protects against type 1 diabetes in the bio-Breeding diabetes-prone rat. Is the gut flora involved in the development of type 1 diabetes?. *Diabetologia*.

[8] Schwartz RF, Neu J, Schatz D, Atkinson MA, Wasserfall C (2007). Comment on: Brugman S et al. (2006) Antibiotic treatment partially protects against type 1 diabetes in the Bio-Breeding diabetes-prone rat. Is the gut flora involved in the development of type 1 diabetes? *Diabetologia* vol. 49, pp. 2105–2108. *Diabetologia*.

[9] Sechi LA, Paccagnini D, Salza S, Pacifico A, Ahmed N, Zanetti S (2008). *Mycobacterium avium* subspecies *paratuberculosis* bacteremia in type 1 diabetes mellitus: an infectious trigger?. *Clinical Infectious Diseases*.

[10] Rosu V, Ahmed N, Paccagnini D (2009). Specific immunoassays confirm association of *Mycobacterium avium* subsp. *paratuberculosis* with type-1 but not type-2 diabetes mellitus. *PLoS ONE*.

[11] Chiodini RJ, Van Kruiningen HJ, Merkal RS (1984). Ruminant *paratuberculosis* (Johne’s disease): the current status and future prospects. *The Cornell veterinarian*.

[12] Ellingson JLE, Anderson JL, Koziczkowski JJ (2005). Detection of viable *Mycobacterium avium* subsp. *paratuberculosis* in retail pasteurized whole milk by two culture methods and PCR. *Journal of Food Protection*.

[13] Naser SA, Ghobrial G, Romero C, Valentine JF (2004). Culture of *Mycobacterium avium* subspecies *paratuberculosis* from the blood of patients with Crohn’s disease. *Lancet*.

[14] Sechi LA, Scanu AM, Molicotti P (2005). Detection and isolation of *Mycobacterium avium* subspecies *paratuberculosis* from intestinal mucosal biopsies of patients with and without Crohn’s disease in Sardinia. *American Journal of Gastroenterology*.

[15] Sechi LA, Gazouli M, Ikonomopoulos J (2005). *Mycobacterium avium* subsp. *paratuberculosis*, genetic susceptibility to Crohn’s disease, and Sardinians: the way ahead. *Journal of Clinical Microbiology*.

[16] Rosu V, Ahmed N, Paccagnini D (2009). Specific immunoassays confirm association of *Mycobacterium avium* subsp. *paratuberculosis* with type-1 but not type-2 diabetes mellitus. *PLoS ONE*.

[17] Di Sabatino A, Paccagnini D, Vidali F (2011). Detection of *Mycobacterium avium* subsp. *paratuberculosis* (MAP)-specific IS900 DNA and antibodies against MAP peptides and lysate in the blood of Crohn’s disease patients. *Inflammatory Bowel Diseases*.

[18] Cossu A, Rosu V, Paccagnini D, Cossu D, Pacifico A, Sechi LA (2011). MAP3738c and MptD are specific tags of *Mycobacterium avium* subsp. *paratuberculosis* infection in type I diabetes mellitus. *Clinical Immunology*.

[19] Cossu D, Cocco E, Paccagnini D (2011). Association of *Mycobacterium avium* subsp. *paratuberculosis* with multiple sclerosis in sardinian patients. *PLoS ONE*.

[20] Cossu D, Masala S, Cocco E Are *Mycobacterium avium* subsp. *paratuberculosis* and Epstein-Barr virus triggers of multiple sclerosis in Sardinia?.

[21] Sechi LA, Rosu V, Pacifico A, Fadda G, Ahmed N, Zanetti S (2008). Humoral immune responses of type 1 diabetes patients to *Mycobacterium avium* subsp. *paratuberculosis* lend support to the infectious trigger hypothesis. *Clinical and Vaccine Immunology*.

[22] Feller M, Huwiler K, Stephan R (2007). *Mycobacterium avium* subspecies *paratuberculosis* and Crohn’s disease: a systematic review and meta-analysis. *Lancet Infectious Diseases*.

[23] Rani PS, Sechi LA, Ahmed N (2010). *Mycobacterium avium* subsp. *paratuberculosis* as a trigger of type 1 diabetes: destination Sardinia, or beyond?. *Gut Pathogens*.

[24] Paccagnini D, Sieswerda L, Rosu V (2009). Linking chronic infection and autoimmune diseases: *Mycobacterium avium* subspecies *paratuberculosis*, SLC11A1 polymorphisms and type-1 diabetes mellitus. *PLoS ONE*.

[25] Masala S, Paccagnini D, Cossu D (2011). Antibodies recognizing *Mycobacterium aviumparatuberculosis* epitopes cross-react with the beta-cell antigen znt8 in sardinian type 1 diabetic patients. *PLoS ONE*.

[26] Wenzlau JM, Juhl K, Yu L (2007). The cation efflux transporter ZnT8 (Slc30A8) is a major autoantigen in human type 1 diabetes. *Proceedings of the National Academy of Sciences of the United States of America*.

